# Geriatric Oral Health for dental and non-dental primary care providers

**DOI:** 10.1093/geroni/igab046.3057

**Published:** 2021-12-17

**Authors:** Maryam Tabrizi

**Affiliations:** The University of Texas School of Dentistry at Houston, Houston, Texas, United States

## Abstract

Age-Friendly Health System is the innovation that provides care to older adults within 4Ms structure comprehensively. The 4Ms structure is the clinicians' guideline for providing treatments for each individual based on what matters most which is the first M. The 4Ms structure creates a person focus approach for the health of the elderly on every visit, as what matters most may change over time based on other health conditions of life situation. We must consider our patients come from all kinds of life walks, based on his/ her lifestyle, health beliefs, and cultural background. Consequently, older adults may define health and wellness differently and they might have different needs, oral health is no exception. However, evaluating both primary care office workflow and hospital workflow is missing the health element of oral care. The current health flow designed by the Institute of Health Improvement (IHI) in a collaborative effort with John A. Hartford may create a larger gap between oral health and overall health. In the light of integrating oral health to the overall health, the best place and most feasible in both primary care and hospital workflow is at the time of Check history for a baseline on 4Ms. This poster will clearly illustrate how oral health can integrate to overall health leading by the non-dental profession who usually take history for a baseline. As oral health is an integrated factor in the health of the geriatric population in the Age-Friendly Health System.

